# Flash Communication: Flexibility of a Biologically Inspired Ligand Framework for Intramolecular C–H Activation

**DOI:** 10.1021/acs.organomet.4c00454

**Published:** 2025-01-17

**Authors:** Jewelianna M. Moore, Yun Ji Park, Alison R. Fout

**Affiliations:** † Department of Chemistry, Texas A&M University, 580 Ross St. College Station, Texas 77843, United States; ‡ School of Chemical Sciences, University of Illinois at Urbana−Champaign, Urbana, Illinois 61801, United States

## Abstract

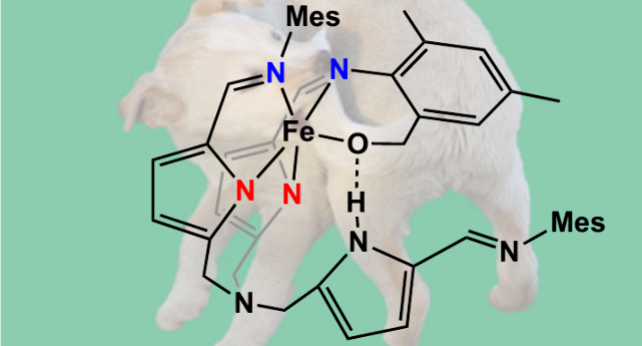

High-valent iron complexes play a crucial role in the oxidation of organic substrates, especially in C–H bond functionalization reactions in biology. This paper investigates the reactivity of nonporphyrin tripodal ligands featuring a secondary coordination sphere, focusing on their prospective ability to stabilize high-valent iron-oxo species. Using NMR spectroscopy and X-ray crystallography, we detail the formation of an Fe(III)-alkoxide complex through intramolecular C–H bond activation, providing insight into the potential transient formation of a high-valent iron-oxo intermediate. While attempts to observe an Fe(IV)-oxo complex were unsuccessful, our findings underscore the significance of the ligand electronic environment in stabilizing reactive iron species for C–H bond activation.

High-valent iron complexes, supported by both heme and nonheme ligands, are pivotal in the oxidation of organic substrates across synthetic and biological systems.^1−3^ In biology, cytochromes P450 are crucial in metabolism and steroid transformation in a variety of organisms.^4^ These compounds are particularly notable for their success in catalytic reactions involving C–H bond functionalization, such as hydroxylation of saturated C–H bonds, oxidation of aromatic substrates, epoxidation of alkenes, and dealkylation reactions.^2,4,5^

The mechanism of cytochrome P450 enzymes is well established to involve the formation of a reactive Fe(IV)-oxo with a porphyrin-based radical cation, commonly referred to as compound I.^6^ Compound I, generated from reaction of the iron(III) center and dioxygen or other oxygen atom sources, such as peroxides, facilitates the insertion of oxygen into the aliphatic position of organic substrates. Mechanistic understanding of C–H activation in hemes has inspired both inorganic and organometallic chemists to utilize reactive high-valent metal-oxo complexes to activate strong bonds.^7−19^

There exists several synthetic high-valent Fe-oxo complexes capable of C–H bond activation in the literature.^1,7,9−15,17−20^ Early research focused on biologically inspired iron porphyrin complexes for olefin epoxidation and alkane hydroxylation.^21,22^ Mechanistic studies of these reactions demonstrated that the Fe(IV)-oxo porphyrin cation radical was responsible for substrate activations, as suggested for cytochrome P450.^23,24^

In addition to heme complexes, bioinspired nonheme high-valent iron complexes have received much attention (Figure 1, select examples).^9,10,20,25−33^ For example, Chang and co-workers reported a high-spin Fe(IV)-oxo supported by a tripodal pyrrolide ligand platform capable of C–H bond activation.^32,34^ At a low temperature, they reported isolation of a high-spin Fe(IV)-oxo complex. When the reaction mixture was warmed to room temperature, the high valent iron complex performed C–H bond activation of the ligand platform, resulting in an Fe(III)-alkoxide complex. In an effort to stabilize generally reactive high-valent Fe-oxo complexes, Borovik and co-workers installed a secondary coordination sphere onto a similar tripodal ligand, enabling the isolation and crystallographic characterization of both an Fe(III)-oxo and an Fe(IV)-oxo at room temperature,^7,30,35−39^ demonstrating the importance of intramolecular interactions within the ligand to stabilize this reactive high valent iron-oxo bond.

**Figure 1:**
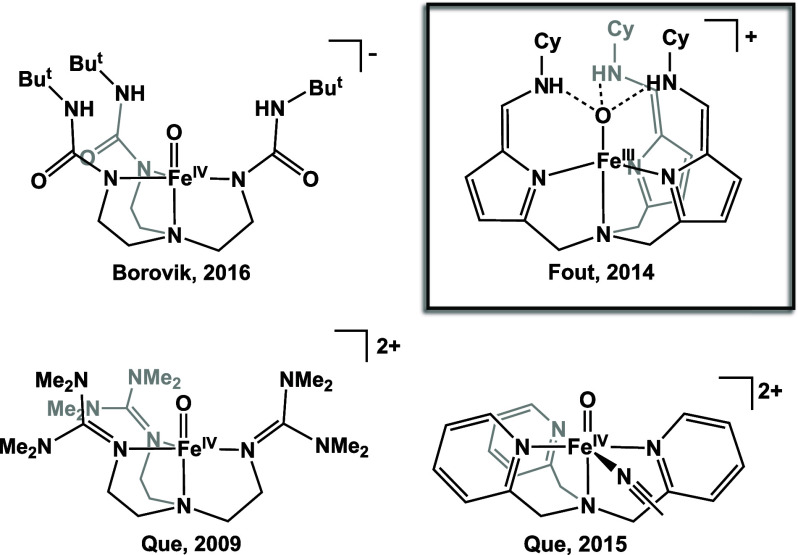
Select tripodal ligand systems able to form high valent iron-oxo species^20,25,36^ compared to previous work from Fout.^44^

Our lab has previously investigated a tripodal ligand featuring a secondary coordination sphere, tris(5-imminopyrrol-2-ylmethyl)amine (H_3_tpa^NR^, R = cyclohexyl (Cy), mesityl (Mes), phenyl (Ph), adamantyl (Ada)).^40−43^ This ligand exhibits unique tautomeric behavior: the pyrrole imine (pi) tautomer provides anionic coordination, while the azafulvene amine (afa) tautomer offers dative coordination to the metal center. Our earlier work focused on the metalated complex [N(afa^Cy^)_3_]Fe(OTf)_2_. The reactivity of [N(afa^Cy^)_3_]Fe(OTf)_2_ toward small molecules such as oxygen, nitrate, perchlorate, and selenate results in the formation of a stable iron(III)-oxo complex, [N(afa^Cy^)_3_]Fe(O)(OTf) (Figure 1, top right).^41,44−48^ In the mesityl variant of the ligand, the sole product observed in oxygen atom transfer reactions is [N(afa^Mes^)_2_(pi^Mes^)]Fe(OH)(OTf), where one ligand arm is deprotonated by the proposed transient Fe-oxo, inducing tautomerization.^41^ In all of these oxygenated complexes, the terminal oxo/hydroxo is stabilized through secondary coordination sphere hydrogen bonds.

The synthesis of a high valent Fe(IV)-oxo was unsuccessful in this ligand framework, presumably because the neutrally coordinated ligand platform is not sufficiently electron rich to stabilize an iron(IV) center. Similar tripodal systems which support high valent iron scaffolds are far more electron donating, typically with anionic coordination to the metal center (Figure 1).^10,30,36−38^ Because we were interested in accessing high-valent iron(IV) complexes with our ligand platform (H_3_[N(pi^Mes^)_3_]), we synthesized an iron(II) complex bearing anionic ligands and reacted it with a two electron oxygen transfer reagent. We expected that the anionic ligand may provide a better electronic environment to access high-valent iron complexes compared to the neutrally bound ligand platform.

To generate an Fe(II) complex where H_3_[N(pi^Mes^)_3_] is anionically bound to the metal center, the ligand was deprotonated using 2.2 equiv of KH. To the solution of the deprotonated ligand, FeCl_2_ was added to produce [N(pi^Mes^)_2_Fe(afa^Mes^)] (**1**, Scheme 1). Characterization by ^1^H NMR spectroscopy confirmed the formation of a new paramagnetic species. IR spectroscopy provided additional insight into the ligand tautomers, showing two C=N stretches at 1578 and 1611 cm^–1^. The C=N stretch at 1578 cm^–1^ is indicative of the iron center being bound to the pyrrole and imine nitrogens of the ligand arms and not in the traditional binding pocket of the ligand.^40,49^ Unfortunately, attempts to crystallize the complex led to formation of the previously published aqua complex [N(afa^Mes^)(pi^Mes^)_2_Fe(OH_2_)] (**2**),^41^ likely due to the heightened reactivity of the electron rich iron center reacting with adventitious water.

**Scheme 1:**
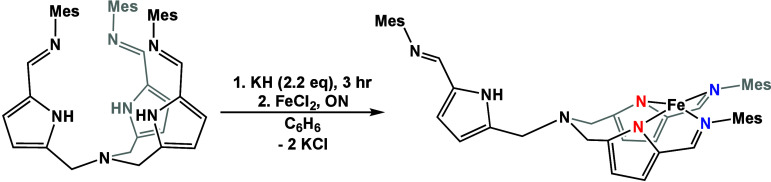
Metalation of H_3_[N(pi^Mes^)_3_] to Form Complex **1**

Oxidation of complex **1** was attempted by the addition of pyridine-*N*-oxide (PyNO) (Figure 2). Given that **1** features a H-bond-donating arm in the secondary coordination sphere, we anticipated stabilization of the oxygenated product. After PyNO was added to **1**, the color of the reaction mixture quickly shifted first from the starting material orange to a transient brown, and finally to green over 24 h. Since two distinct color changes are indicative of multiple events, the reaction was monitored by UV–visible spectroscopy at −40 °C over several hours and ^1^H NMR spectroscopy at room temperature over a 24 h period to elucidate the reaction progress. While intermediates were observed in the absorbance spectra, no absorbances were consistent with a short-lived high-valent iron complex (Figure S9). In the ^1^H NMR spectrum, new paramagnetic resonances appeared accompanied by a decrease in the resonances corresponding to complex **1** by 20 min. After 7 h, the transient paramagnetic resonances began to fade, and after 24 h, new broad paramagnetic resonances were observed at 45.7 and 18.2 ppm (Figure 3).

**Figure 2:**
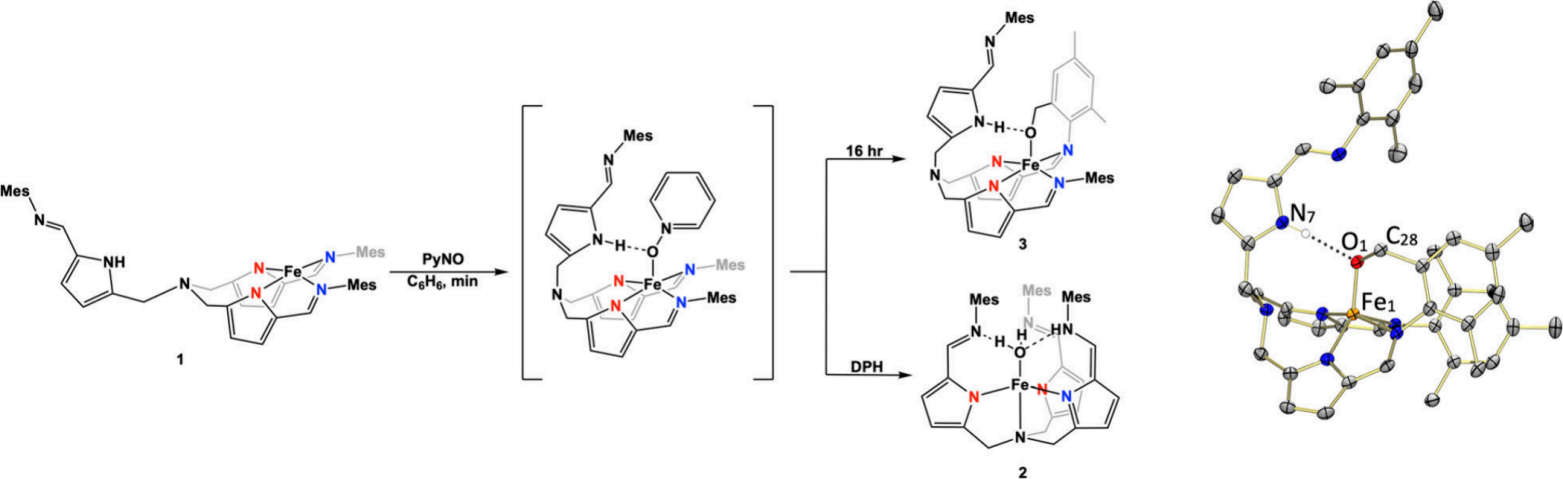
Reactivity of **1** with PyNO, showcasing intramolecular C*–*H activation and intermolecular N*–*H activation pathways. Crystal structure of **3** is shown on right (H atoms removed for clarity, 50% probability ellipsoids).

**Figure 3:**
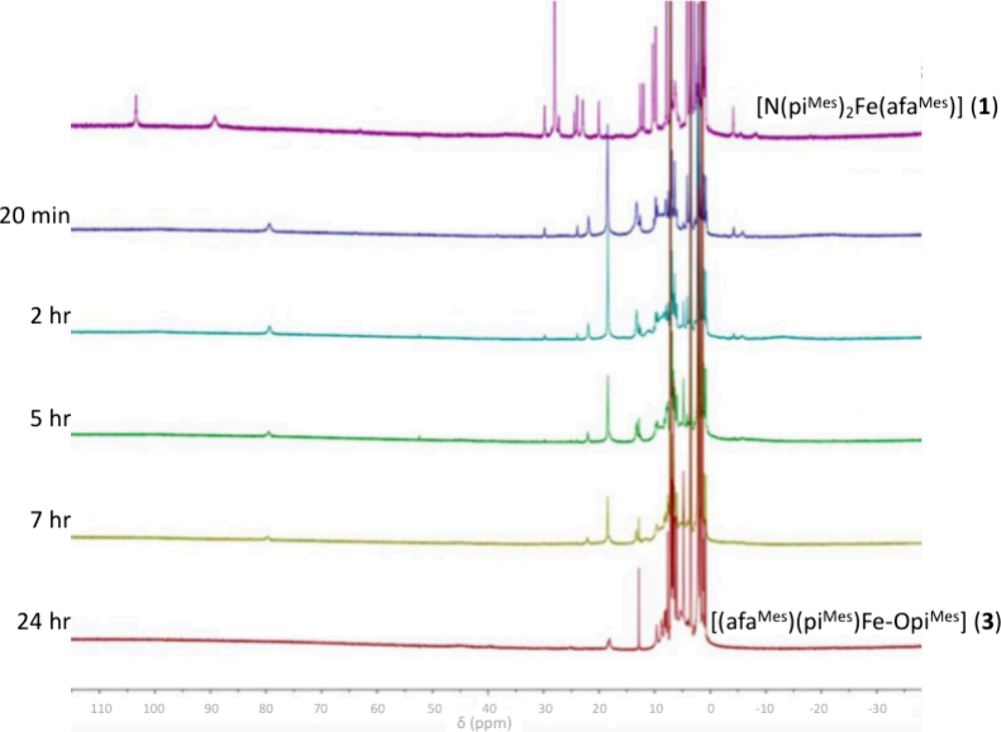
Stacked ^1^H NMR spectra depicting the conversion of complex **1** to complex **3** over time with the proposed intermediate **2** shown after 20 min.

To further understand these results, structural characterization of the final green product was accomplished by X-ray diffraction of crystals grown from a concentrated hexane solution at −35 °C. X-ray analysis revealed that the iron center was in a distorted square pyramidal geometry, bound to four pyrrole imine nitrogen atoms in two ligand arms. Excitingly, the axially bound oxygen was appended to one of the mesityl methyl groups, resulting from an intramolecular benzylic C–H bond oxidation of one of the pendant methyl groups to give [(afa^Mes^)(pi^Mes^)Fe-Opi^Mes^] (**3,**Figure 2). Analogous intramolecular C–H bond activation was observed by Chang and co-workers when [tpa^Ph^Fe^II^]^+^ was reacted with trimethylamine N-oxide to form a low temperature stable Fe(IV)-oxo. When warmed to room temperature, the analogous iron alkoxide complex was formed, where the bond length of the reported Fe–O bond (1.903(5) Å) is comparable to the Fe–O bond length (1.843(2) Å) of complex **3**.^32,34^ As expected, the Fe-Opi^Mes^ adduct is stabilized through H-bonding from the pyrrole hydrogen, acting as a secondary coordination sphere.

Based on the solid-state structure of **3**, we propose the diamagnetic resonances in the ^1^H NMR spectrum similar to the free ligand corresponds to the intact arm of the ligand platform positioned relatively far from the paramagnetic iron center (Figure S3), while the paramagnetic resonances correspond to the rest of the ligand including the C–H bond activated methyl group. The IR spectrum was consistent with the X-ray crystal structure, as it contains three unique C=N stretches (1584 cm^–1^, 1608 cm^–1^, and 1622 cm^–1^) corresponding to three chemically inequivalent ligand arms. We propose the two arms directly bound to the iron center are inequivalent due to one of the arms being oxygenated and ligated to iron at the mesityl methyl position. While we were not able to isolate the transient brown product, we propose PyNO forms an adduct with the metal complex within 20 min, as observed by ^1^H NMR spectroscopy. Comparable to Chang’s work,^32,34^ we hypothesize an oxygen atom transfer event from PyNO to the iron center, followed by C–H bond cleavage of the ortho-methyl of the mesityl group to form the resulting Fe(III)-alkoxide compound (Scheme 1, top path).^50^

Further characterization by UV–vis spectroscopy confirmed the formation of the high-spin Fe(III) complex. In the UV–vis spectrum of complex **3**, an absorption band at 629 nm (ε = 570 M^–1^cm^–1^) was detected which did not appear in the spectrum of the starting compound **1.** This feature resembles previously reported high-spin Fe(III) complexes,^50,51^ suggesting **3** is a high-spin Fe(III) complex.

Unfortunately, attempts to isolate and characterize reactive intermediates proved to be unsuccessful. However, the facile generation of the C–H-activated oxygenated product, Fe(III)-alkoxide, from oxygen transfer reagents may imply the formation of a potent iron-centered oxidant such as an Fe(IV)-oxo. Moreover, addition of diphenylhydrazine (DPH) to the mixture of [N(pi^Mes^)_2_Fe(afa^Mes^)] and PyNO solution resulted in clean formation of previously published [N(afa^Mes^)(pi^Mes^)_2_Fe(OH_2_)] as identified by ^1^H NMR spectroscopy (**2,**Figure 2, bottom path).^41^ This result suggests that the generation of the Fe(IV)-oxo complex would subsequently react twice with the N–H bonds of DPH to generate complex **2**.

Although we were unable to isolate a high-valent Fe(IV) complex, the formation of either the intramolecular C–H activated Fe(III)-alkoxide complex **3** or the diphenylhydrazine activated complex **2** indicates the transient presence of a reactive high valent iron species. This reactivity is unique to the anionically coordinated tautomer of the ligand, which can be easily accessed by modifying the metalation strategy. This study highlights the importance of the ligand’s electronic environment in stabilizing reactive iron complexes and suggests that future efforts to achieve high-valent iron species may benefit from further modifications to the ligand platform.
