# Supplementary material for: Flash Communication: Flexibility of a Biologically Inspired Ligand Framework for Intramolecular C–H Activation
Source: Organometallics. 2025 Jan 17;44(3):472–6. doi: 10.1021/acs.organomet.4c00454 (PMC11816012; doi:10.1021/acs.organomet.4c00454)
Supplement: Supplementary file 1 — om4c00454_si_001.pdf [file om4c00454_si_001.pdf]

Supporting Information

Flash Communication: Flexibility of a Biologically Inspired Ligand Framework for Intramolecular C-H Activation

Jewelianna M. Moore,[a] Yun Ji Park,[b] and Alison R. Fout[a*]

[a]*Department of Chemistry, Texas A&M University, 580 Ross St. College Station, Texas 77843, USA*

[b]*School of Chemical Sciences, University of Illinois at Urbana-Champaign, Urbana, IL, 61801.*

Email: fout@tamu.edu

Table of Contents

General Considerations....S2
Materials and Methods....S2
Physical Measurements....S2
Experimental Procedures....S2
Synthesis of Metalated Complexes....S2
Figures S1-S9....S3
Crystallographic Structure....S8
Crystallographic Parameters....S9
References....S9

General Considerations:

Materials and Methods.

To avoid contact with oxygen and water, all manipulations were carried out under an atmosphere of nitrogen in an MBraun or Vigor inert atmosphere drybox or using standard Schlenk techniques. Solvents for air- and moisture-sensitive manipulations were dried and deoxygenated using a Glass Contour System (SG Water USA, Nashua, NH) and stored over 4Å molecular sieves purchased from Strem prior to use. Celite 545 (J. T. Baker) was heated to 150°C under dynamic vacuum for 24 h prior to use in the drybox. All reagents were purchased from commercial sources and used as received unless otherwise noted. 1,2-Diphenylhydrazine was purchased from Oakwood Chemical and recrystallized from diethyl ether and hexane under an inert atmosphere prior to use. $H_3[N(pi^{Mes})_3]$ and was synthesized according to literature procedure.[1] NMR solvent (benzene-d_6) was purchased from Cambridge Isotope Laboratories, degassed, and stored over 4Å molecular sieves prior to use.

Physical Measurements.

NMR spectra were recorded at ambient temperature on a Varian spectrometer operating at 400 or 500 MHz (^{1}H NMR) and referenced to the peak of the residual solvent (δ parts per million and J in Hz). Solid-state infrared spectra were measured using a PerkinElmer Frontier FT-IR spectrophotometer equipped with a KRS5 thallium bromide/iodide universal attenuated total reflectance accessory. Ultraviolet-visible (UV-vis) spectroscopy was performed on an Agilent Technologies Cary Series UV-vis NIR 5000 spectrometer. All samples were prepared in a drybox containing a dinitrogen atmosphere in quartz cuvettes with a 1 cm path length and capped with a rubber septum.

Experimental Procedures:

Synthesis of Metal Complexes.

Preparation of $[N(pi^{Mes})_2Fe(afa^{Mes})]$ (1). $H_3[N(pi^{Mes})_3]$ (0.070 g, 0.1 mmol) was deprotonated by addition of 2.2 equivalent KH (0.009 g, 0.22 mmol) in approximately 10 mL of benzene solution. After it was stirred for three hours at room temperature, the mixture was filtered through Celite to remove excess KH. Addition of deprotonated ligand to the $FeCl_2$ (0.013 g, 0.1 mmol) slurry in 1 mL of benzene resulted in a color change from colorless to yellow. After stirring overnight until all $FeCl_2$ was consumed, the reaction mixture was filtered through Celite to remove KCl and the solvents were removed under reduced pressure to give an orange powder in quantitative yield. The resulting orange compound was assayed by ^{1}H NMR spectroscopy. IR: ν = 1578 cm^{-1} (C═N, strong), 1611 cm^{-1} (C=N, weak). HRMS-ESI: calculated $[M+H]^+$: 744.3472, found: 744.3456.

Oxidation of $[N(pi^{Mes})_2Fe(afa^{Mes})]$ to form $[(afa^{Mes})(pi^{Mes})Fe\text{-}Opi^{Mes}]$ (3). To the benzene solution of $[N(pi^{Mes})_2Fe(afa^{Mes})]$ prepared in situ (described above, 11 mL benzene), pyridine N-oxide (0.010 g, 0.1 mmol) was added as a white solid. The mixture was stirred overnight resulting in green-brown solution, after which time solvent was removed under reduced pressure. The resulting powder was dissolved in hexanes (10mL) and filtered through Celite to remove brown byproduct. The solvent was removed in vacuo to reveal a green powder in 67% yield. Green crystals suitable for X-ray analysis were grown from a concentrated hexanes solution at -35°C. IR: ν = 1581, 1608, 1622 cm^{-1} (C═N, medium). HRMS-ESI: calculated $[M+H]^+$: 759.3343, found: 759.3311.

Oxidation of $[N(pi^{Mes})_2Fe(afa^{Mes})]$ with DPH present to form $[N(afa^{Mes})(pi^{Mes})_2Fe(OH_2)]$ (2). To the benzene solution of $[N(pi^{Mes})_2Fe(afa^{Mes})]$ prepared in situ (described above), pyridine N-oxide (0.010 g, 0.1 mmol) and diphenylhydrazine (0.018 g, 0.1 mmol) were added as white solids. The mixture was stirred overnight in the dark, resulting in an orange solution, after which time solvent was removed under reduced pressure. The resulting compound was assayed by ^1H NMR spectroscopy, revealing the formation of $[N(afa^{Mes})(pi^{Mes})_2Fe(OH_2)]$[1] (**2**) in quantitative yield.

Control Reaction of DPH and PyNO. Pyridine N-oxide (0.010 g, 0.1 mmol) and diphenylhydrazine (0.018 g, 0.1 mmol) were added to 10 mL of benzene. The mixture was stirred overnight in the dark, after which time solvent was removed under reduced pressure. The resulting compound was assayed by ^1H NMR spectroscopy, showing no reaction between the two reagents.

*Figure S1. ^1H NMR spectrum of $[N(pi^{Mes})_2Fe(afa^{Mes})]$ (**1**) (C_6D_6).*

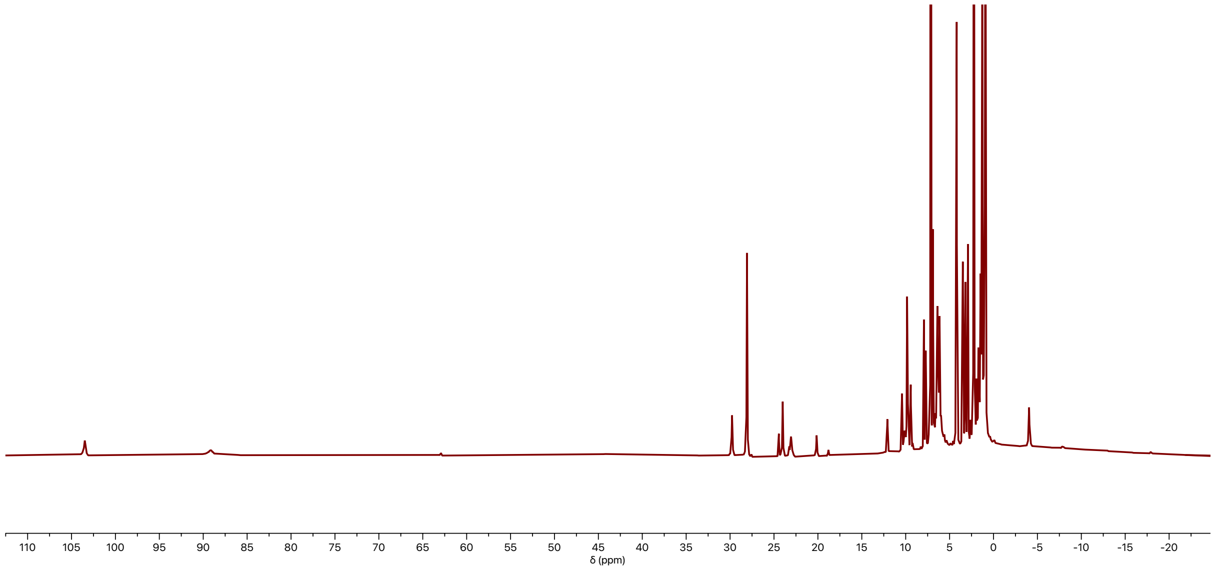

*Figure S2. ¹H NMR spectrum of crystalline [(afa^{Mes})(pi^{Mes})Fe-Opi^{Mes}] (**3**) (C_6D_6).*

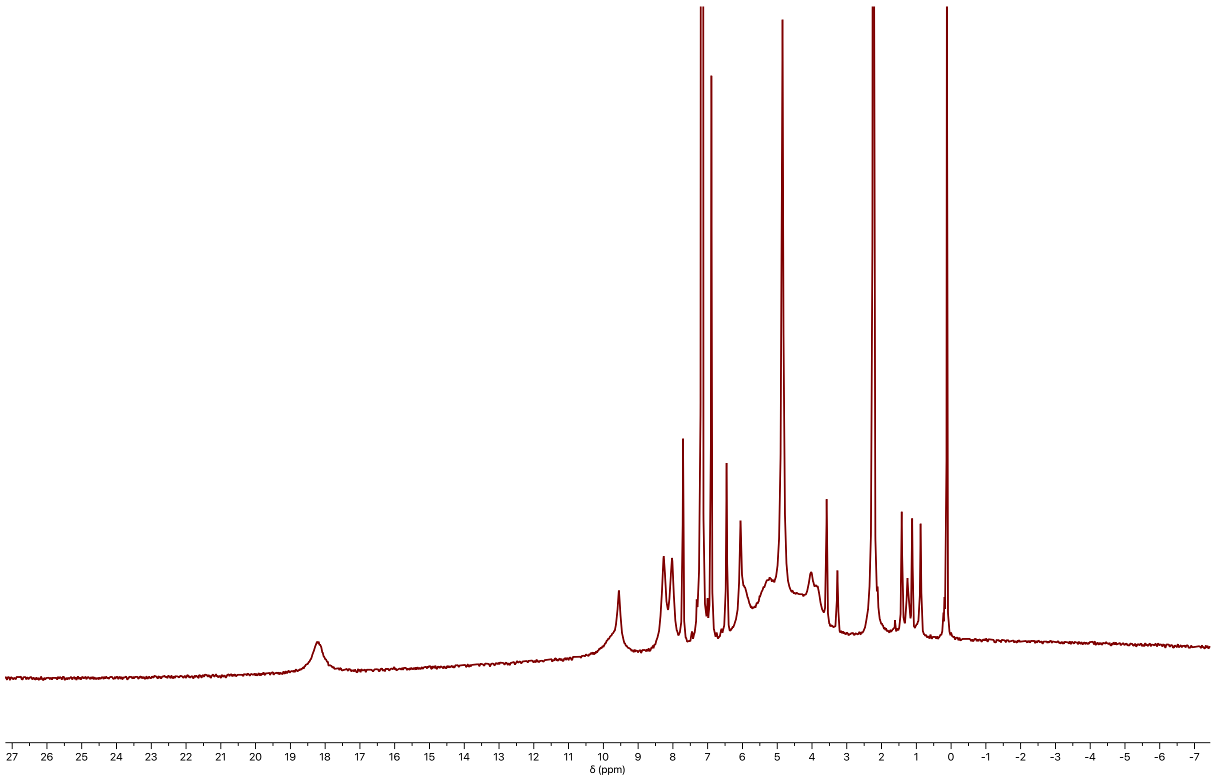

*Figure S3. ¹H NMR spectrum of diamagnetic region of crystalline **3** showing similarities to free ligand (C_6D_6).*

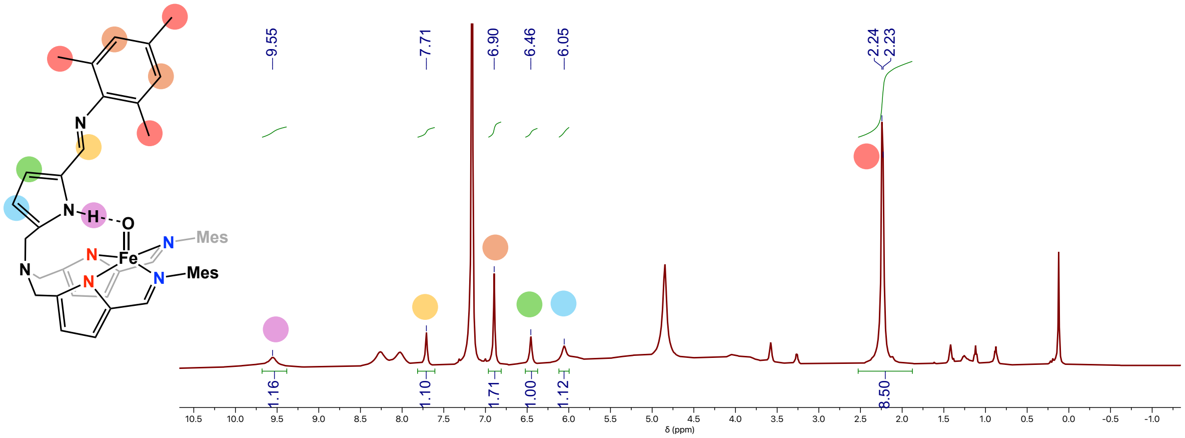

*Figure S4. ^{1}H NMR spectrum of DPH present oxidation of **1** to form **2** (C_6D_6).*

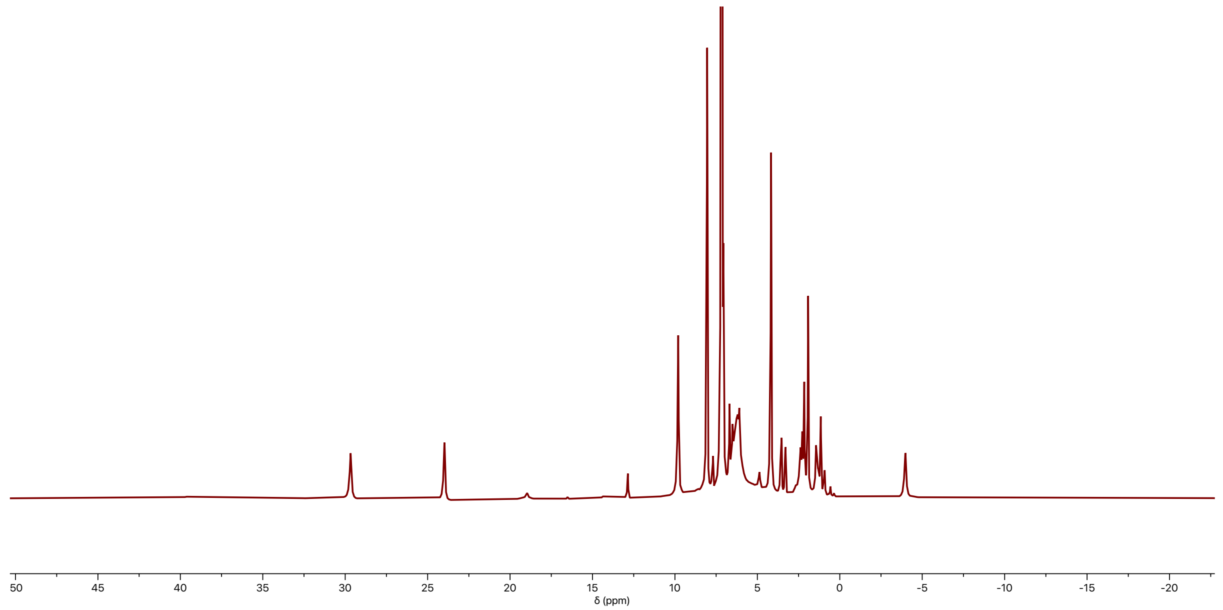

Figure S5. ^{1}H NMR spectrum of the control reaction of DPH and PyNO (C_6D_6).

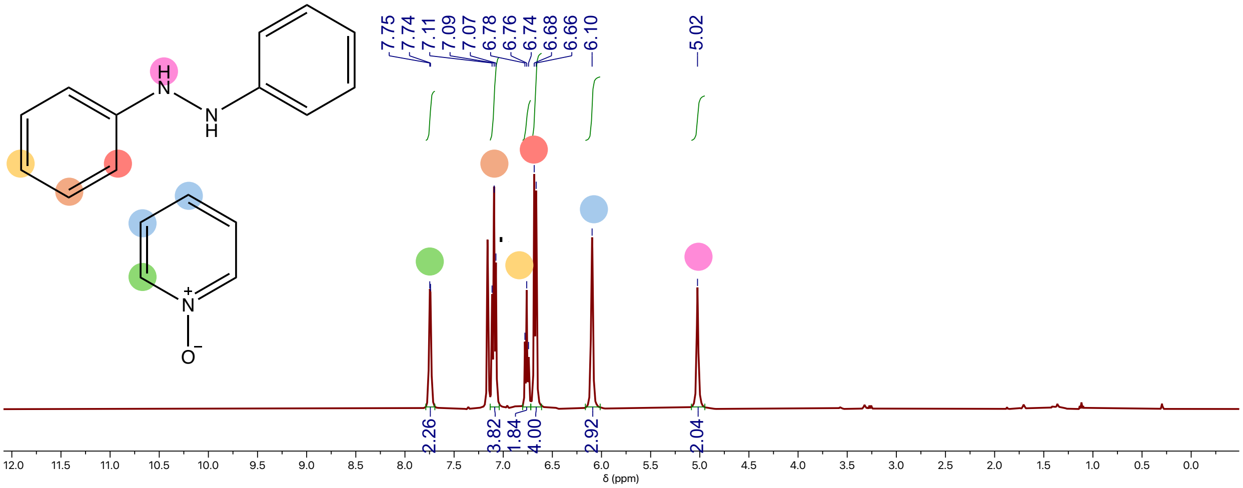

*Figure S6. Infrared spectrum of complex **1**.*

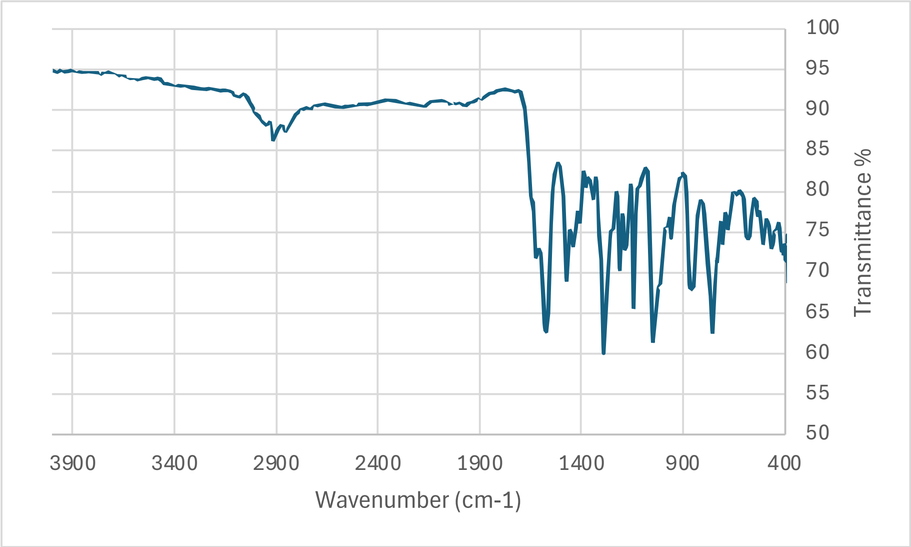

*Figure S7. Infrared spectrum of complex **3** with inlay of C=N stretching region.*

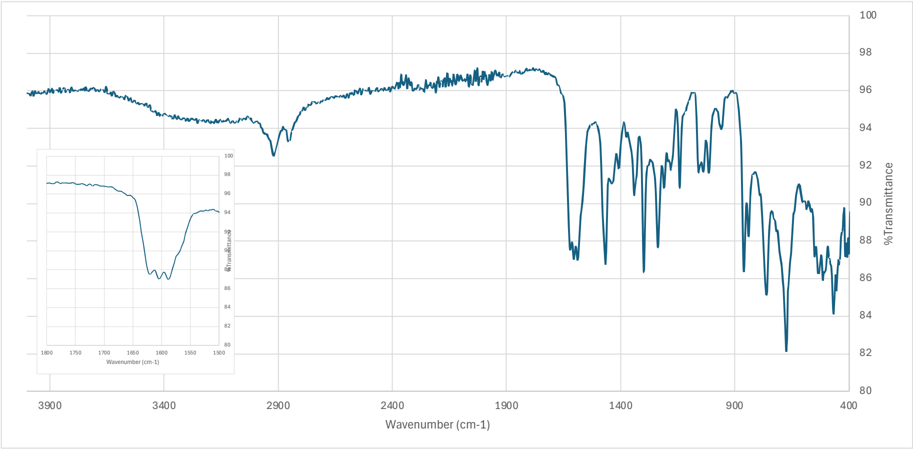

*Figure S8. Absorbance spectra of complex **1** before and after reaction with PyNO (1.0 mM in benzene).*

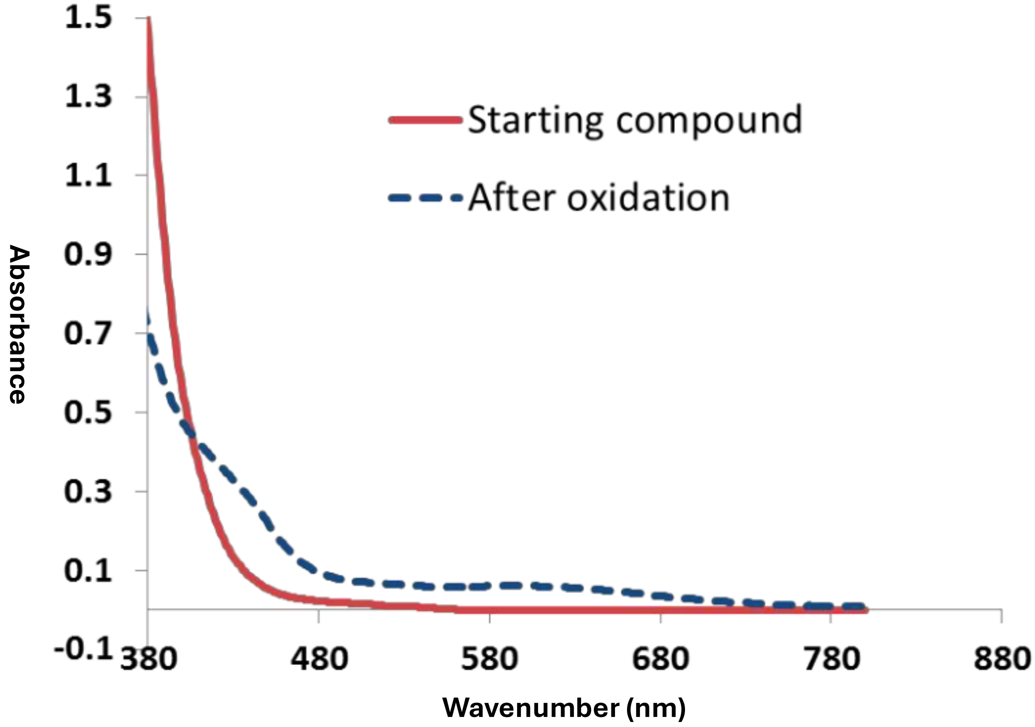

*Figure S9. Absorbance spectra showing region of interest of complex **1** and PyNO (1.0 mM in THF) at -40°C recorded every 2 min for one hour then every 10 min for 3 hours. Increasing base line is an artifact of the cold temperature UV-Vis spectrometer.*

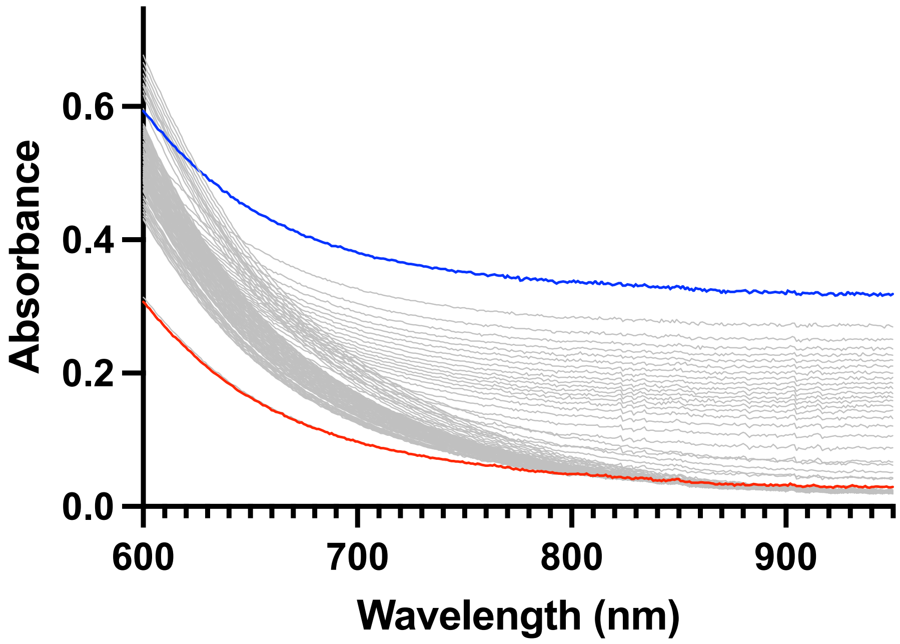

Crystallographic Structure and Selected Bond Lengths

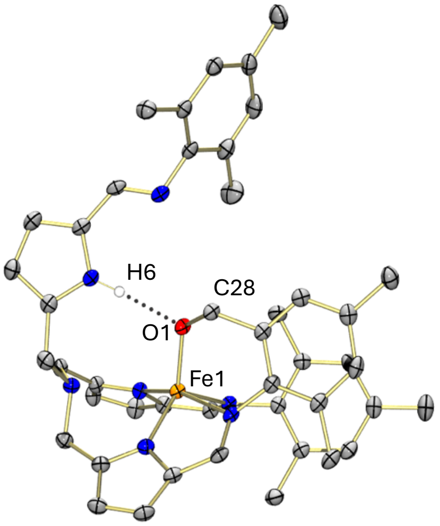

	Selected Bond Lengths
Fe1-O1	1.8531(10) Å
O1-H6	1.9896(13) Å
O1-C28	1.427(2) Å

Crystallographic parameters

Formula	$C_{50.76}H_{61.44}FeN_7O$
$D_{calc.}$/ g cm^{-3}	1.240
m/mm^{-1}	0.380
Formula Weight	841.48
Color	brown
Shape	block-shaped
Size/mm^3	0.36×0.13×0.07
T/K	99.99
Crystal System	triclinic
Space Group	*P*-1
a/Å	15.6488(7)
b/Å	16.9115(9)
c/Å	18.6910(10)
a/°	81.733(2)
b/°	75.400(2)
g/°	70.618(2)
V/Å^3	4505.7(4)
Z	4
Z'	2
Wavelength/Å	0.71073
Radiation type	MoK$_a$
Q_{min}/°	2.349
Q_{max}/°	26.444
Measured Refl's.	161287
Indep't Refl's	18540
Refl's I≥2 *s*(I)	15413
R_{int}	0.0445
Parameters	1102
Restraints	231
Largest Peak	0.355
Deepest Hole	-0.364
GooF	1.016
wR_2 (all data)	0.0965
wR_2	0.0898
R_1 (all data)	0.0472
R_1	0.0362

References

(1) Park, Y. J.; Peñas-Defrutos, M. N.; Drummond, M. J.; Gordon, Z.; Kelly, O. R.; Garvey, I. J.; Gullett, K. L.; García-Melchor, M.; Fout, A. R. Secondary Coordination Sphere Influences the Formation of Fe(III)-O or Fe(III)-OH in Nitrite Reduction: A Synthetic and Computational Study. *Inorg. Chem.* **2022**, *61* (21), 8182–8192.
